# S100A9 Activates the Immunosuppressive Switch Through the PI3K/Akt Pathway to Maintain the Immune Suppression Function of Testicular Macrophages

**DOI:** 10.3389/fimmu.2021.743354

**Published:** 2021-10-26

**Authors:** Zun Pan Fan, Mei Lin Peng, Yuan Yao Chen, Yu Ze Xia, Chun Yan Liu, Kai Zhao, Hui Ping Zhang

**Affiliations:** ^1^ Institute of Reproductive Health, Tongji Medical College, Hua Zhong University of Science and Technology, Wuhan, China; ^2^ Tongji Medical College, Hua Zhong University of Science and Technology, Wuhan, China

**Keywords:** S100A9, macrophage polarization, testicular macrophage, immune suppression, orchitis

## Abstract

Macrophages are functionally plastic and can thus play different roles in various microenvironments. Testis is an immune privileged organ, and testicular macrophages (TMs) show special immunosuppressive phenotype and low response to various inflammatory stimuli. However, the underlying mechanism to maintain the immunosuppressive function of TMs remains unclear. S100A9, a small molecular Ca^2+^ binding protein, is associated with the immunosuppressive function of macrophages. However, no related research is available about S100A9 in mouse testis. In the present study, we explored the role of S100A9 in TMs. We found that S100A9 was expressed in TMs from postnatal to adulthood and contributed to maintaining the immunosuppressive phenotype of TMs, which is associated with the activation of PI3K/Akt pathway. S100A9 treatment promotes the polarization of bone marrow-derived macrophages from M0 to M2 *in vitro*. S100A9 was significantly increased in TMs following UPEC-infection and elevated S100A9 contributed to maintain the M2 polarization of TMs. Treatment with S100A9 and PI3K inhibitor decreased the proportion of M2-type TMs in control and UPEC-infected mouse. Our findings reveal a crucial role of S100A9 in maintaining the immunosuppressive function of TMs through the activation of PI3K/Akt pathway, and provide a reference for further understanding the mechanism of immunosuppressive function of TMs.

## Introduction

Macrophage has been classically divided into two polarization programs *in vitro* (1), (1) classically activated macrophages or M1, which are usually stimulated by TH1 type cytokines, resulting in anti-bacterial and pro-inflammatory properties and (2) alternatively activated macrophages or M2, which are induced by TH2 type cytokines, resulting in anti-inflammatory, pro-repair phenotype. Generally, M1 play a major role in the early stage of infection to resist pathogens, while M2 play a major role in the recovery stage to promote tissue repair (2). Polarized macrophages display different patterns of glucose metabolism, M1-type macrophages are characterized by rapid glycolysis ability, while M2-type macrophages are mainly characterized by oxidative phosphorylation (3). Macrophage polarization is modulated by local microenvironment, and different polarization status affects the outcome of inflammation (4).

As first-line immune defense cells against inflammation in the testis, testicular macrophages (TMs) show unique tolerance to pathogens and play a key role in maintaining the immune privileged state of the testis and spermatogenesis (5). Tissue-resident TMs are usually M2 under physiological conditions, exhibited immuno-suppressive feature by secreting high levels of IL10 or TGFβ, and showed an inactivation of NF-κB signaling during inflammatory stimulation (6). The immunosuppression phenotype of rat TMs is associated with high levels of immunomodulatory molecules in testicular interstitial fluid, such as testosterone, prostaglandin, and corticosterone (7). Other molecules such as activin A, 25-HC may also affect the phenotype of testicular macrophages (8). However, the mechanism of regulating the immunosuppressive function of TMs remains unclear.

Notably, S100A9, a Ca^2+^ binding protein (9), which is mainly derived from macrophages, regulates immune function by affecting the phenotype of macrophages in neonatal mouse intestinal tissue under physiological conditions. Intestinal tissues from S100a9^-/-^ neonatal mouse showed altered phenotype of colonic lamina propria macrophages in colon with reduced IL-10 and TGF-β (secreted by M2-type macrophage) mRNA level, downregulated expression of CX3CR1 protein (macrophage marker), and fewer regulatory T cells (10). In the blood of human newborn, S100A9, which is higher than adult under physiological conditions, inhibits the ability of fast-glycolysis (M1-type feature) in neonatal cord blood-derived macrophages, causing impaired ability of reaction to pathogens (11). Therefore, S100A9 is closely associated with the activation of M2 macrophages under physiological condition. However, the role of S100A9 in TMs has not been studied.

PI3K/Akt signaling is a critical pathway that converges metabolic and inflammatory signals to regulate macrophage response and modulate activation phenotype (12). The activation of PI3K signaling is a critical molecular switch that controls macrophage immune suppression function in cancer and other disorders (13); PI3K signaling through Akt and m-TOR inhibits NF-κB activation, leading to an immunosuppressive function during inflammation (13). Significant upregulation of S100A9, accompanied by activation of PI3K/Akt pathway, has been found in various diseases related to immune escape, such as colitis (14), breast cancer (15), and psoriasis (16). However, whether S100A9 affects the polarization of TMs by activating PI3K/Akt pathway remains unknown.

In the present study, we tested the proportion of M2-type TMs in mouse from postnatal to adulthood and S100A9 expression in TMs. The effect of S100A9 on the polarization of mouse TMs was verified *via* the intervention of tasquinimod (S100A9 inhibitor). The function of S100A9 *in vitro* on the polarization of bone marrow-derived macrophages was determined by exogenous supplementation of S100A9. The effect of S100A9 on the PI3K/Akt pathway was evaluated by the knockdown or overexpression of S100A9 in Raw264.7 mouse macrophage cell line. Finally, we verified that elevated S100A9 following UPEC-infection contributed to maintain the M2 polarization of TMs through the activation of PI3K/Akt pathway.

## Materials and Methods

### Animals

C57BL/6 mice (2-, 4-, 6-, 8- and 10-week-old) (2week, n=15; 4week, n=12; 6week, n=32; 8week, n=35; 10week, n=8) were obtained from the Animal Centre of Tongji Medical College. All animals were housed and handled in strict accordance with the guidelines approved by the Animal Care and Use Committee of Tongji Medical College, Huazhong University of Science and Technology (No. 2019_S326).

### Bacterial Culture and Animal Treatment

Urinary pathogenic *E. coli* (UPEC) strain CFT073 was propagated, and UPEC-induced experimental orchitis model was prepared as previously described (17). The testis, epididymis, and vas deferens of male C57BL/6 mice were fully exposed after anaesthesia. Approximately 8×10^4^ per 10 µl of UPEC saline suspension was injected into the vas deferens proximal to the caudal epididymis. The sham group was injected with the same amount of PBS. Ligation was performed at the injection site to prevent the spread of infection. The S100A9 protein (RD, 2065-S9-050) was dissolved in PBS. Tasquinimod (S100A9 inhibitor, ABR-215050, MCE, 254964-60-8) was dissolved in dimethyl sulfoxide (DMSO) and diluted with PBS to adjust the concentration of DMSO to 3%. On day 2 post-infection, ABR-215050 (S100A9 inhibitor, 20 mg/kg) and LY294002 (50 mg/kg) were injected into the upper, middle and lower pole of UPEC-infected mouse testis (at a two-day interval, twice totally). The concentration of S100A9 was adjusted and injected about 10-15ul into each testicle. DMSO (3%) was injected into the testis as control.

### Identification of Testis Infection and Sample Collection

The animals were sacrificed on the 7th day after infection. Testicular tissues were collected for subsequent experiments. A small piece of testicular tissue was taken from control and UPEC-infected groups, homogenized and diluted in 1ml of sterile PBS. 100ul testicular homogenate was evenly coated on LB solid medium and incubated at 37°C overnight. The CFU of UPEC and control group was compared the next day.

### Western Blot Analysis

Total protein was extracted from macrophages by radio-immunoprecipitation assay (RIPA) buffer containing cocktail protease inhibitor and phosphor–protease inhibitor (Servicebio, G2006/G2007). Protein concentration was determined using BCA kit (Thermo Fisher, 23225). Equal amounts of protein (5–25 µg) were loaded in appropriate concentration of sodium dodecyl sulphate (SDS)–polyacrylamide gel electrophoresis and transferred into polyvinylidene fluoride (PVDF) membrane. The membranes were blocked with 5% bovine serum albumin and incubated at 4°C overnight with the following primary antibodies: rabbit anti-S100A9 (1:1,000, abcam,242945), rabbit anti-PI3K (1:1,000, CST, 4257), rabbit anti-AKT (1:1,000, CST, 4691), rabbit anti-p-AKT (1:1,000, CST, 4060), rabbit and rabbit anti-β-actin (1:1,000, CST, 4970). The membranes were then incubated with goat anti-rabbit secondary antibody (1:4,000, Biosharp) for 2 h at room temperature. Protein bands were detected using Femto-light chemiluminescence kit (Omni-ECL, SQ201) and chemiluminescence-gel imaging system ECL (Board, USA). The results were analysed with help of the Image J software. Densitometry values were normalized to total β-actin.

### Single-Cell Suspension of Testicular Interstitial Cells

The animals were sacrificed after anesthesia, and their testicles were removed. Interstitial cells were isolated as previously described (17). After careful dissection of tunica albuginea, the de-capsulated testes were digested with 1 mg/ml collagenase I (Sigma, USA) and 10 μg/ml DNase I in a shaking water bath at 34°C for 15 min. When the tubules were dispersed, digestion was terminated. The tubules were then filtered with a 200-mesh sieve, and the filtrate was centrifuged at 800 ×*g* for 5 min. RBC was removed with erythrocyte lysate. After resuspension with PBS, the single-cell suspension of testicular interstitial cells was obtained.

### Flow Cytometry

Interstitial cells were collected and stained according to the manufacturer’s instructions. The following antibodies were used: anti-mouse CD16/32 (Biolegend, 156604), Zombie-APC/CY7 (Biolegend, 423106), FITC anti-mouse CD45 (Biolegend, 103108), PE anti-mouse F4-80 (Biolegend, Clone: BM8, 123110), PE/cyanine5 anti-mouse CD86 (Biolegend, 105016), and Brilliant Violet 605™ anti-mouse CD206 (Biolegend, 141721). The labelled cells were detected using a flow cytometer (Cytek Aurora or BD LSRII). Data were analysed using FlowJo software (Version 10.4.0, USA).

### Immunofluorescence Staining

The testicular tissue was fixed in 4% paraformaldehyde, subjected to gradient dehydration, embedded with Tissue-tek OCT (AKURA, 4583), snap frozen in liquid nitrogen, and cut into 5 µm-thick sections. After antigen retrieval, the slides were permeabilized with 0.5% Tritox-100, blocked with 5% BSA, and then incubated with rabbit anti-S100A9 (1:1,000, Abcam, 242945) and Alexa Fluor^®^ 594 anti-mouse F4/80 (1:100, Biolegend, 123140) as primary antibodies. The secondary antibody was goat anti-rabbit Alexa Fluor 488(1:100). The nuclei were stained with DAPI, and images were acquired using fluorescence microscope (Mshot, MF43).

### Immunohistochemistry

The testis was fixed in 4% paraformaldehyde, embedded with paraffin wax, and cut into 5 µm-thick sections. The slides were dewaxed in xylene and rehydrated in gradient alcohol solution. After antigen retrieval, the slides were added with 3% hydrogen peroxide to prevent endogenous peroxidase formation and then blocked with 3% BSA. The expression of S100A9 was detected by incubation with primary rabbit anti-S100A9 (1:1,000, Abcam, 242945) and appropriate secondary antibodies.

### Magnetic-Activated Cell Sorting for TMs

The interstitial cells were counted, resuspended in 0.5–1 ml auto MACS running buffer (Miltenyi,130-091-221-1), and incubated for 15 min at 4°C in the dark with Anti-F4/80-MicroBeads (Miltenyi, 130-110-443) according to the manufacturer’s instructions. The cells were washed and centrifuged at 300 ×*g* for 10 min. Cellular suspension was loaded into the LS separation column previously rinsed with 3 ml of MACS running buffer and attached to MACS separator (Miltenyi, 130-042-301). The positive fraction (F4/80 positive macrophage) was attached to the column, while the negative fraction was eluted through the column (discarded). The magnetically labelled cells were flushed immediately by firmly pushing the plunger into the column. The cells were identified through FCM by using PE anti-mouse F4-80 (Biolegend, Clone: BM8, 123110). The purity of TMs was about 85%.

### RNA Isolation and qPCR

The total RNA of BMDMs and TMs was extracted using TRIzol reagent (Invitrogen, 15596018, China) according to the manufacturer’s recommendation, and then transcribed into cDNA by using HiScript II Q RT SuperMix for qPCR (11123 ES, China). qRT-PCR amplification was performed using Hieff qPCR SYBR Green Master Mix (112101ES03, China) on LightCycler^®^96 (Roche, Basel, Switzerland). The prime sequences are shown in **Table 1**. Relative mRNA levels were normalized to β-actin expression and determined using the 2^-ΔΔCT^ method.

**Table 1:** Primers used for qPCR in this study.

Mouse	Sense 5′-3′	Anti-sense 5′-3′
IL10	TTCTTTCAAACAAAGGACCAGC	GCAACCCAAGTAACCCTTAAAG
TGFβ1	CCAGATCCTGTCCAAACTAAGG	CTCTTTAGCATAGTAGTCCGCT
S100A9	CACAGTTGGCAACCTTTATGAA	TCATACACTCCTCAAAGCTCAG

### Plasmid Construction and Transfection

S100A9 over-expressing vector, shRNA-expressing vector and their negative control plasmid were purchased from Genomeditech (Shanghai, China). The entire S100A9 sequence was included in the S100A9-overexpression vector. BamHI and XhoI sites were inserted in the overexpression vector PGMLV-CMV-MCS-EF1-ZsGreen1-T2A-Puro through a double-restriction enzyme digest. Three shRNAs targeting S100A9 were constructed by pGMLV-SC5 RNAi lentivirus vector. Plasmid titers were detected using Nanodrop2000 (Thermo Scientific, USA) and transfected into RAW 264.7 cell lines by using High-gene Transfection reagent (Abclone, RM09014) according to the manufacturer’s instruction. The sequences and primers are shown in Supplementary material (**Supplementary Table S1**).

### Statistical Analysis

All data were presented as mean and standard deviation (mean ± SD). GraphPad Prism 7 (GraphPad Software, USA) was used for data analysis through analysis of variance and Dunnett’s multiple comparisons test. P-value < 0.05 indicated significant difference.

## Results

### S100A9 Is Associated With Immunosuppressive Phenotype of TMs

**Figure 1:**
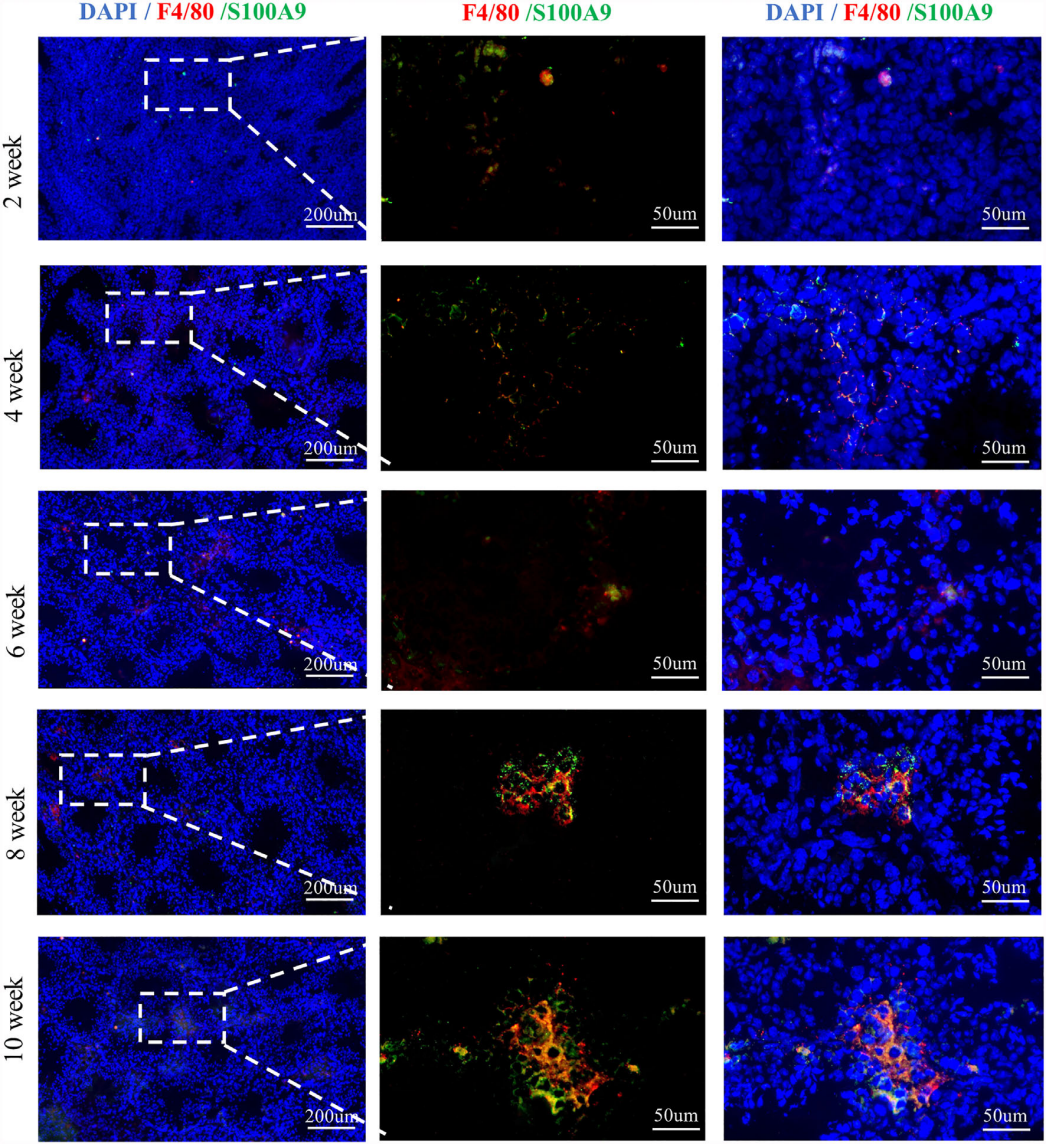
S100A9 expressed in testicular macrophages of mouse from weeks 2 to 10. Double immunofluorescence staining of S100A9 and mouse macrophage marker (F4/80) in testis from weeks 2 to 10 (scale bar: 200 and 50μm).

We first tested the expression of S100A9 in the testis. We found that S100A9 was expressed in the TMs of mouse from postnatal to adulthood based on F4/80 and S100A9 dual immunofluorescence localization (**Figure 1** and **Supplementary Figure S1**).

CD68 was expressed in most TMs of rats (5) and distinguished by CD68^+^CD163^-^(M1) and CD68^+^CD163^+^(M2). In mouse, no definite markers are available to identify TM1 and TM2; core macrophage markers such as CXC3R1, F4/80, CD11b, and AIF, are expressed in most TMs in mouse (18). CD80, CD86 and cytokines such as IL1, TNF-a are usually secreted by M1 (1, 19–22), while CD163 and CD206 were used to mark M2 in mouse (21–24).

**Figure 2:**
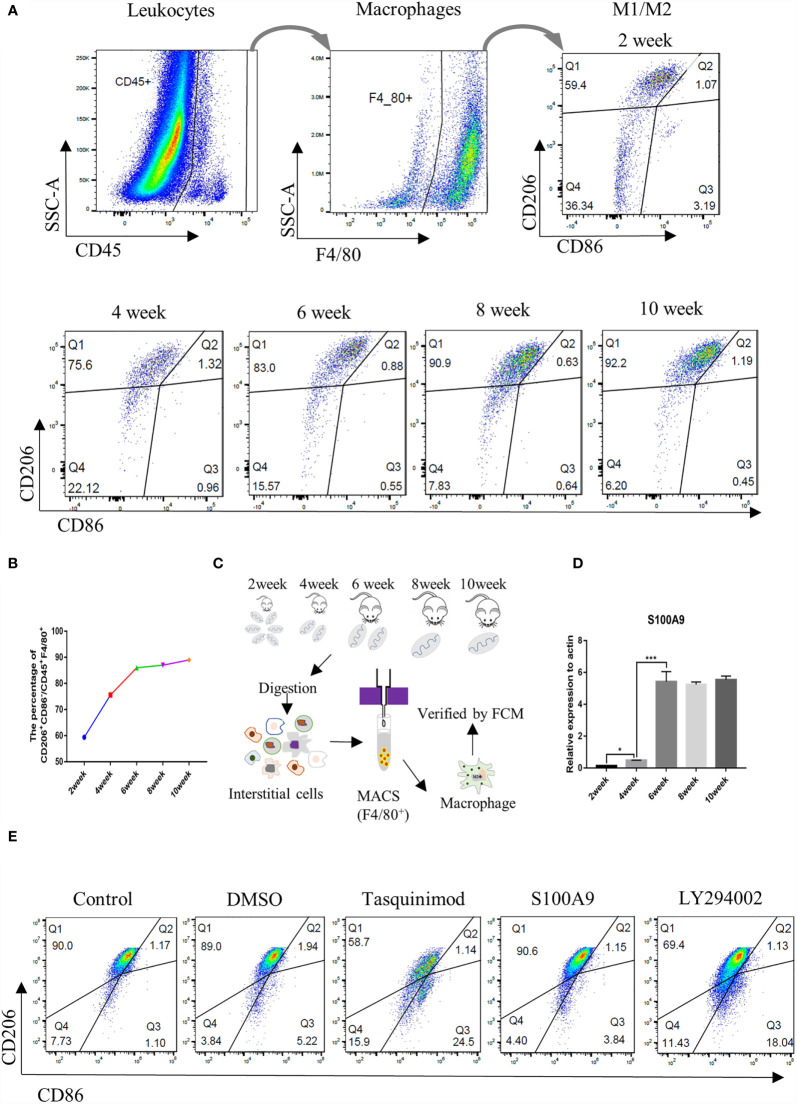
Ratio of M2 TMs and S100A9 mRNA expression increases with the development of mouse testis. **(A)** Gating strategy that describes CD45^+^F4/80^+^ mouse testis fraction and the distinction of M2 (CD206^+^CD86^-^) and M1 (CD206^-^CD86^+^) population, represented by Q1 and Q3 respectively (2week, *n*=4/group; 4-6week, *n*=3/group; 8-10week, *n*=2/group). **(B)** Percentage of M2Φ in different age of TMs by FCM. **(C)** Flow chart of preparation for TMs, testes from weeks 2 to 10 were digested and interstitial cells were incubated with F4/80 magnetic beads (2week, *n*=8/group; 4-6week, *n*=6/group; 8-10week, *n*=4/group). TMs were obtained by magnetic cell sorting (MACS), and the purity was verified by flow cytometry for subsequent experiment. **(D)** Relative mRNA expression of S100A9 at different ages of TM (2week, *n*=6/group; 4-6week, *n*=5/group; 8-10week, *n*=4/group). **(E)** Percentage of M2Φ in adult mouse testis (8week, *n*=2/group) under physiological conditions decreased with the treatment of S100A9 inhibitor (tasquinimod) or PI3K inhibitor (LY294002), compared with the control group. DMSO and S100A9 groups were used as control. Data represent the mean ± standard error (n=2-3). Significant differences between the two groups are indicated as *P < 0.05, ***P < 0.001; comparisons between groups using one-way ANOVA followed by Dunnett’s multiple comparisons test.

Thus, in the present study, we observed the change of TMs in mouse by flow cytometry by using CD45^+^ (leukocyte marker), F4/80^+^ (macrophage marker), CD206^+^ (M2 marker), and CD86^+^ (M1 marker). Testicular interstitial cells were collected, and the percentage of CD45^+^ leukocyte, CD45^+^F4/80^+^macrophage, CD45^+^F4/80^+^CD206^+^CD86^-^ M2, and CD45^+^F4/80^+^CD206^-^CD86^+^ M1 groups were analysed using flow cytometry (**Figure 2A** and **Supplementary Figure S2**). We found that the total number of leukocytes and macrophages in testis was increased with the development of testis (**Supplementary Figure S3**); the proportion of M2-TMs increased gradually from postnatal (2 weeks) to adulthood (≥ 6 weeks) and became stabilized at approximately 90% after the sixth week (**Figure 2B**). Almost all adult testicular macrophages expressed CD206, and most of them were M2 after 6 weeks. TMs were collected using magnetic-activated sorting by using F4/80 magnetic beads and the purity was identified by FCM (**Figure 2C** and **Supplementary Figure S4**). Then, we compared the mRNA expression of S100A9 (**Figure 2D**) in TMs from week 2 to week 10. Notably, the change trend of S100A9 in TMs was consistent with the increasing trend of M2 macrophages in testis. Therefore, S100A9 may be related to maintaining the immunosuppressive phenotype of testicular macrophages.

### S100A9 Maintains the M2 Polarization State of Testicular Macrophages

To further clarify whether S100A9 plays a role in regulating TMs polarization, we injected tasquinimod (S100A9 inhibitor) and S100A9 into testis. DMSO was used as the control group. The percentage of M1 (marked as CD45^+^F4/80^+^CD206^-^CD86^+^) and M2 (marked as CD45^+^F4/80^+^CD206^+^CD86^-^) in each group was detected by FCM analysis (**Figure 2E** and **Supplementary Figure S5**). No obvious change was observed in DMSO and S100A9 group compared with the control group. Treatment with tasquinimod (S100A9 inhibitor) substantially decreased the M2:TM ratio from 90.0% to 58.7%, and the M1:TM ratio increased from 1.10% to 24.5% between the control and tasquinimod group. This result was similar with the treatment by LY294002 (PI3K inhibitor). M2:TM ratio decreased from 90.0% to 69.4%, and the M1:TM ratio increased from 1.10% to 18.04% between the control and LY294002 group. Therefore, S100A9 contributes to maintain the immunosuppressive phenotype of TMs through the activation of PI3K/Akt pathway.

### S100A9 Treatment Promotes the Polarization of Bone Marrow-derived Macrophages to M2 *In Vitro*

**Figure 3:**
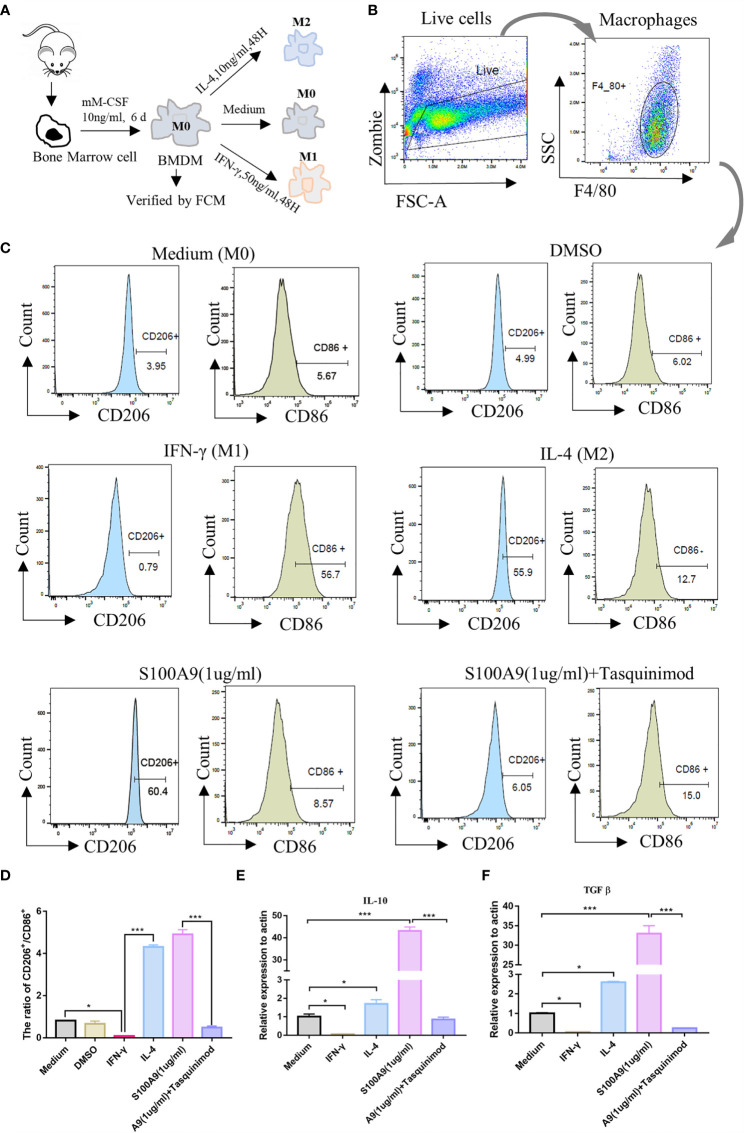
S100A9 treatment promotes the polarization of bone marrow-derived macrophages to M2 *in vitro*. **(A)** Mouse (6week, *n*=4/group) bone marrow single cell suspension was collected through a 70μm filter, and removed red blood cells by red cell lysis solution. BMDMs (M0) were induced with mM-CSF (10 ng/ml, 6 days) in RPMI1640 medium. Further polarization was achieved by adding 50 ng/ml IFN-γ (M1), 10 ng/ml IL-4 (M2), DMSO (0.3%), S100A9 (1 µg/ml) and S100A9 (1 µg/ml)+tasquinimod (10 µmol/ml) for 48 more hours. **(B, C)** Immunotyping was performed by flow cytometric analysis to detect live cells, macrophage surface markers (F4/80^+^), M1-MΦ marker (CD86^+^), and M2-MΦ marker (CD206^+^). **(D)** The ratio of CD206^+^ and CD86^+^ in different groups. **(E, F)** Relative mRNA expression of IL10 and TGFβ in different groups. Data represent the mean ± standard error(*n*=3–5). *P < 0.05, ***P < 0.001. comparisons between groups using one-way ANOVA followed by Dunnett’s multiple comparisons test.

We isolated and cultured mouse bone marrow-derived macrophages (BMDMs, designated as M0) *in vitro* (**Figure 3A**) and stimulated them with S100A9 (1 µg/ml), IL-4 (10 ng/ml, designated as M2) and IFN-γ (50 ng/ml, designated as M1) for 48 h, respectively. DMSO (0.3%) was used as the control. Tasquinimod (10 µmol/ml, inhibitor of S100A9) was compared with the S100A9 group. M1/M2 subpopulation in each group was distinguished by the expression of M1 (CD45^+^F4/80^+^CD86^+^) and M2 (CD45^+^F4/80^+^CD206^+^), as shown in **Figures 3B, C** and **Supplementary Figure S6**. We obtained an extremely similar trend (the ratio of M2/M1) between IL4 and S100A9 group, and the ratio of M2/M1 was reversed with the treatment of tasquinimod (**Figure 3D**). We also found that S100A9 significantly increased the mRNA expression of IL10 and TGFβ (**Figures 3E, F**) in the S100A9 group, and treatment with tasquinimod significantly decreased the expression of IL10 and TGFβ, which were usually secreted by M2-type macrophages. Thus, S100A9 stimulation promoted the polarization of BMDMs from M0 to M2 *in vitro*.

### S100A9 Activates the PI3K/Akt Pathway in Raw 264.7 Macrophages

**Figure 4:**
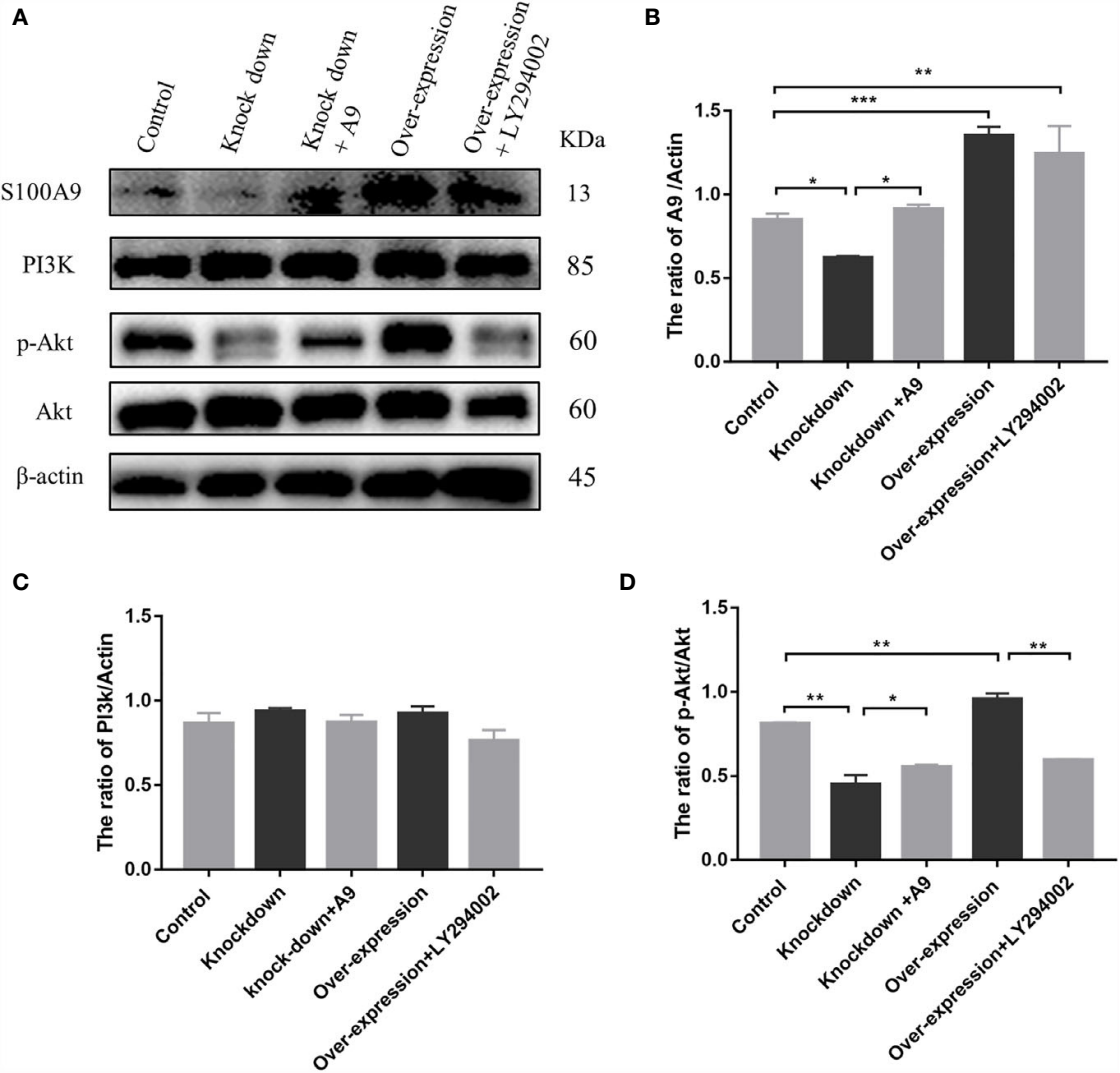
S100A9 treatment activates PI3K/Akt pathway in Raw 264.7 macrophage cells. **(A)** Western blot results and quantification data of S100A9, PI3K, p-Akt, and Akt expression in different groups. Quantification data of Western blot analysis results for the ratio of **(B)** S100A9/Actin, **(C)** PI3K/Actin, and **(D)** p-Akt/Akt. Data are presented as mean ± standard error (*n*=2–3); *P < 0.05, **P < 0.01, ***P < 0.001; comparisons between groups using one-way ANOVA followed by Dunnett’s multiple comparisons test.

To verify whether S100A9 can activate the immunosuppression switch of macrophages to maintain the immunosuppressive phenotype by activating PI3K/Akt pathway, we overexpressed and knocked down S100A9 in mouse RAW264.7 macrophage cell line. PI3K inhibitor (LY294002) was used to inhibit the activation of the PI3K/Akt pathway. Then, the expression levels of S100A9, PI3K, Akt, and p-Akt in the five groups, namely, the control, S100A9 knockdown (S100A9-KD), S100A9 overexpression (S100A9-OE), S100A9-KD+S100A9, and S100A9-OE+LY294002 group, were tested (**Figure 4A** and **Supplementary Figure S7**). In comparison with the control group, the expression of S100A9 significantly increased after overexpression, decreased in S100A9-KD group, and then reversed after exogenous supplementation (**Figure 4B**). The expression of PI3K showed no obvious change in each group (**Figure 4C**). In comparison with the control group, the ratio of p-Akt/Akt was significantly upregulated in the S100A9-OE group. Treatment with LY294002 significantly attenuated the upregulation ratio of p-AKT/Akt (**Figure 4D**). The ratio of p-Akt/Akt was significantly downregulated in S100-KD group, but the ratio then significantly increased with the supplementation of S100A9 (**Figure 4D**). Hence, S100A9 overexpression activates the PI3K/Akt pathway.

### Expression of TM-Derived S100A9 Increase in UPEC-Induced Orchitis

**Figure 5:**
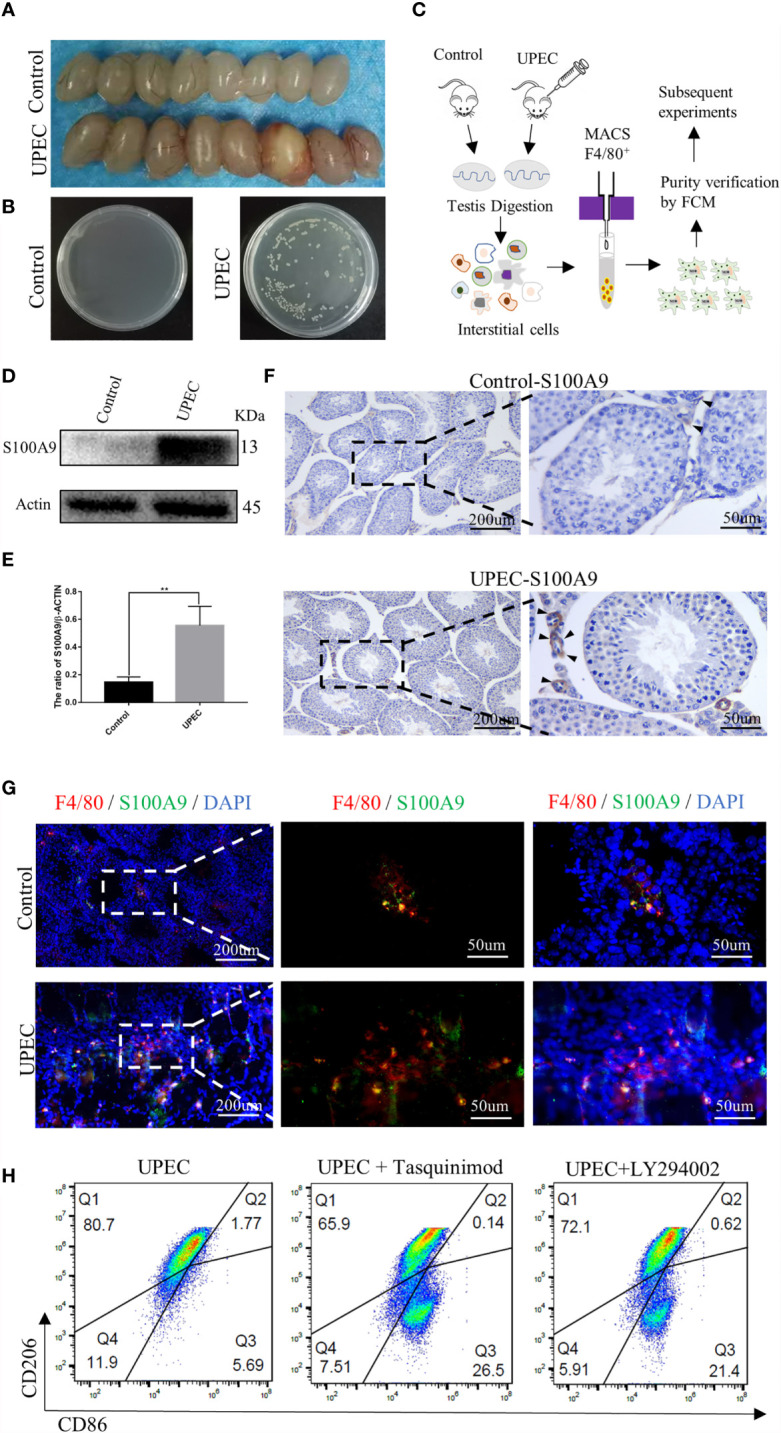
S100A9 expression increased in TMs and treatment with tasquinimod or LY294002 attenuated the ratio of M2-TMs following UPEC-induced orchitis. **(A)** Comparison of testis between the control and UPEC infection groups (8week, n=4/group). **(B)** The growth of the colonies after homogenization and plating of testis tissues in the control and UPEC groups (n=4/group). **(C)** Flow chart of preparation for TMs, testes from control and UPEC group were digested and interstitial cells were incubated with F4/80 magnetic beads (8week, n=4/group). TMs were obtained by magnetic cell sorting (MACS), and the purity was verified by flow cytometry for subsequent experiment. **(D, E)** Western blot analysis results and quantification data of S100A9 expression in TMs of control and UPEC group (8week, *n*=4/group). Data represent the mean ± standard error. **P < 0.01. **(F)** Immunohistochemistry staining result of S100A9 in the control and UPEC group testis (8week) at the 7th day after infection. Black arrows showed the expression of S100A9 (scale bar: 200, 50 μm). **(G)** Double immunofluorescence staining of S100A9 and mouse macrophage marker (F4/80) in the testis (8week) of control and UPEC group (scale bar: 200, 50 μm). **(H)** Flowcytometric analysis of total TMs (8week, *n*=2/group) was marked as CD45^+^F4/80^+^. Percentage of M2Φ in testis decreased with the treatment of S100A9 inhibitor (tasquinimod) or PI3K inhibitor (LY294002), compared with the UPEC group.

We successfully established the mouse UPEC model according to previous methods (17). The testes of the two groups (**Figure 5A**) were mashed and smeared on a bacterial plate. Compared with the control group, the generation of strains was visible to the naked eye in UPEC group (**Figure 5B**). The TMs of mouse were collected using F4/80 magnetic beads at the seventh day after UPEC infection to detect the expression of S100A9 (**Figure 5C**). The purity of TMs was verified by FCM (**Supplementary Figure S4**). Western blot (**Figures 5D, E** and **Supplementary Figure S8**) analysis and immunohistochemistry (**Figure 5F**) showed that the expression of S100A9 in TMs was significantly increased after UPEC infection. Double-immunofluorescence staining (**Figure 5G** and **Supplementary Figure S8**) of F4/80 and S100A9 showed increased expression of S100A9 in testis following UPEC infection.

### Inhibition of S100A9 or Blocking of the PI3K/Akt pathway Reduce the Proportion of M2-TMs Following UPEC-Induced Orchitis

Although the expression of S100A9 increased following UPEC infection, the percentage of M1-TM (marked as CD45^+^F4/80^+^CD86^+^CD206^-^) increased from 1.10% to 5.69%, and M2-TM (marked as CD45^+^F4/80^+^CD206^+^CD86^-^) decreased from 90% to 80.7% (**Figures 2E**, **5H**). To further identify S100A9 contributes to M2 polarization of TMs through the activation of PI3K/Akt pathway, we injected tasquinimod (S100A9 inhibitor) or LY294002 (PI3K inhibitor) individually into the mouse testis two days after UPEC infection. On the seventh day after infection, the percentage of M1 and M2 in each group was detected by FCM analysis (**Figure 5H** and **Supplementary Figure S5**). Treatment with tasquinimod further decreased the ratio of M2-TM from 80.7% to 65.9% but increased the ratio of M1-TM from 5.69% to 26.5% between the UPEC and UPEC + tasquinimod group. Treatment with LY294002 (inhibitor of PI3K) also attenuated M2-TM from 80.7% to 72.1% but increased the ratio of M1-TM from 5.69% to 21.4% between UPEC and UPEC+LY294002 group (**Figure 5H**). S100A9 inhibitor and PI3K inhibitor both decreased the ratio of M2-TMs. Therefore, the elevated S100A9 following UPEC infection contributed to maintain the M2 polarization of TMs through the activation of PI3K/Akt pathway. This finding is consistent with the phenomenon we observed in physiological conditions.

## Discussion

Overall, this study found a new role of S100A9 in maintaining the immunosuppression phenotype of TMs. We elucidated the mechanism by which S100A9 can maintain the immunosuppressive phenotype *via* the activation of PI3K/Akt pathway, an immunosuppressive switch in macrophages. In addition, elevated S100A9 following UPEC-infection contributes to maintaining the M2-type polarization of TMs. Our findings enriched the understanding of the immunosuppressive phenotype of TMs and pave the way to explore the fate of testicular inflammation.

Based on our results, S100A9 expressed in TMs of mouse. The mRNA expression of S100A9 and the proportion of M2-TMs gradually increased with testicular development and remained relatively stable after sperm maturation (at 6 weeks later). Almost all adult testicular macrophages showed M2 types under physiological conditions, and this property may help in maintaining the immune-privileged state of testis to ensure that the antigens on the mature sperm surface are not attacked by themselves. S100A9 inhibitor significantly downregulated the proportion of M2-TMs from 90.0% to 58.7%, this confirming the crucial role of S100A9 in maintaining the M2 polarization state of testicular macrophages.

Bone marrow-derived macrophages induced by macrophage colony-stimulating factor were usually used for *in vitro* studies of macrophage polarization because of its unchanged original “activation” status of macrophages (25). We found that S100A9 promoted the polarization of bone-marrow-derived macrophages from M0 to M2 *in vitro* and increased the mRNA expression of IL-10 and TGFβ. This result is consistent with the findings in myelodysplastic syndromes (MDS) (26), in which S100A9 lead abnormal increase of myeloid-derived suppressor cells and suppressive cytokines IL-10 and TGF-β and finally contributed to immunosuppression, inflammation, and cancer. This result also explains why testicular macrophages secreted a large amount of IL-10 during inflammation stimuli and showed an immunosuppression phenotype (7).

Considering the inability of primary testicular macrophages proliferation *in vitro*, we overexpressed and knockdown S100A9 in Raw 264.7 macrophage cell line and confirmed that S100A9-OE activates the immunosuppressive switch of macrophages through the activation of PI3K/Akt pathway. Finally, we validated this phenomenon by inhibiting S100A9 and PI3K pathways in UPEC-induced orchitis. The elevated S100A9 in orchitis helps in maintaining the M2 polarization of TMs and attenuated the ratio of M1, which usually represent the ability of macrophages to resist bacteria. Similarly, the inhibition of S100A9 or PI3K pathways reduced the M2 ratio of TMs in control and UPEC group. Our results are consistent with the results of a study about fetal cord blood-derived macrophages. The high level of S100A9 in fetal cord blood altered the phenotype of fetal cord blood macrophages by preventing m-TOR expression and inhibiting glycolysis (11). Therefore, an attenuated response to inflammatory stimuli was observed. Rapid glycolysis is a characteristic of M1 macrophages. m-TOR, as a key sensor of nutrient status, is the downstream signal of the PI3K/Akt pathway, and is inhibited by activating the PI3K/Akt axis (12). Moreover, the phenotype of colonic lamina propria macrophages in S100A9^-/-^ mice was altered, and the level of IL10 and TGF-β secreted by M2 macrophages decreased; the number of CX3CR1 protein (macrophage marker) was also decreased (10), which is consistent with our results. Overall, our results revealed a new role of S100A9 in maintaining the immune suppression phenotype of TMs.

Besides, S100A9 contributes to maintain the polarization of M2 TMs, which may impair their ability to resist bacterial in the early stage of infection, but it may also promote the transformation of acute inflammation to chronic (2) and accelerate the repair of tissues in the late stage of infection. In human orchitis, persistent chronic inflammatory response following acute orchitis is often accompanied by a large number of immune cell infiltration in testis and severe fibrosis around seminiferous tubules (27). Among infertile men with genital tract infection, up to 25% of infertile men experience local inflammatory reaction and immune cell infiltration by testicular biopsies (28). The positive effect of targeting S100A9 has been observed in many chronic inflammatory diseases with abnormal tissue repair, such as pulmonary fibrosis (29), renal fibrosis (30), atherogenesis (31), and systemic sclerosis phenotype (32). Whether S100A9 mediates testicular inflammation during the long recovery process following acute infection needs further study.

**Figure 6:**
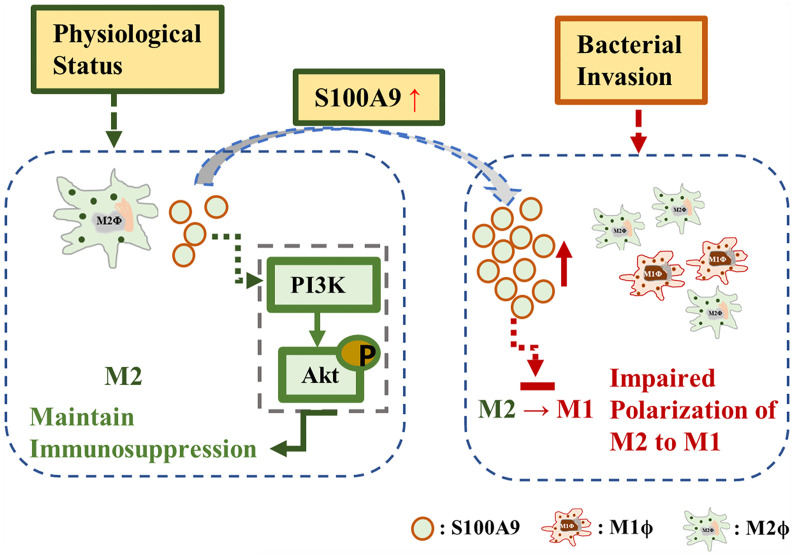
S100A9 mechanism. S100A9 contributed to maintain the M2 polarization of testicular macrophages through the activation of PI3K/Akt pathway under physiological condition. During inflammation, the elevated S100A9 weakened the polarization of testicular macrophages from M2 to M1.

In conclusion, our study reveals a previously unrecognized function of S100A9 in TMs, in which it maintains the immune suppression phenotype of TMs *via* the activation of the PI3K/Akt pathway for a better understanding of the special immunosuppressive function of TMs (**Figure 6**). In orchitis, elevated S100A9 contributes in maintaining the M2 polarization of TMs, which partly explain the immunosuppressive phenotype of TMs to bacterial stimulation. Whether S100A9 can affect the inflammatory outcome in testis and promote tissue repair by affecting the polarization of macrophages to M2 worth further study.

## Data Availability Statement

The original contributions presented in the study are included in the article/**Supplementary Material**. Further inquiries can be directed to the corresponding author.

## Ethics Statement

The animal study was reviewed and approved by Committee of Tongji Medical College, Huazhong University of Science and Technology.

## Author Contributions

ZF and YC designed the study. ZF and MP performed the experiment. YX assembled the data. ZF and CL wrote the paper. KZ and HZ revised manuscript. All authors read and approved the submitted manuscript version.

## Funding

This work was supported by the National Natural Science Foundation of China (Grant No. 8187061007).

## Conflict of Interest

The authors declare that the research was conducted in the absence of any commercial or financial relationships that could be construed as a potential conflict of interest.

## Publisher’s Note

All claims expressed in this article are solely those of the authors and do not necessarily represent those of their affiliated organizations, or those of the publisher, the editors and the reviewers. Any product that may be evaluated in this article, or claim that may be made by its manufacturer, is not guaranteed or endorsed by the publisher.
