# Supplementary material for: S100A9 Activates the Immunosuppressive Switch Through the PI3K/Akt Pathway to Maintain the Immune Suppression Function of Testicular Macrophages
Source: Front Immunol. 2021 Oct 26;12:743354. doi: 10.3389/fimmu.2021.743354 (PMC8576360; doi:10.3389/fimmu.2021.743354)
Supplement: Supplementary file 1 [file DataSheet_1.docx]

**Supplementary materials**

**
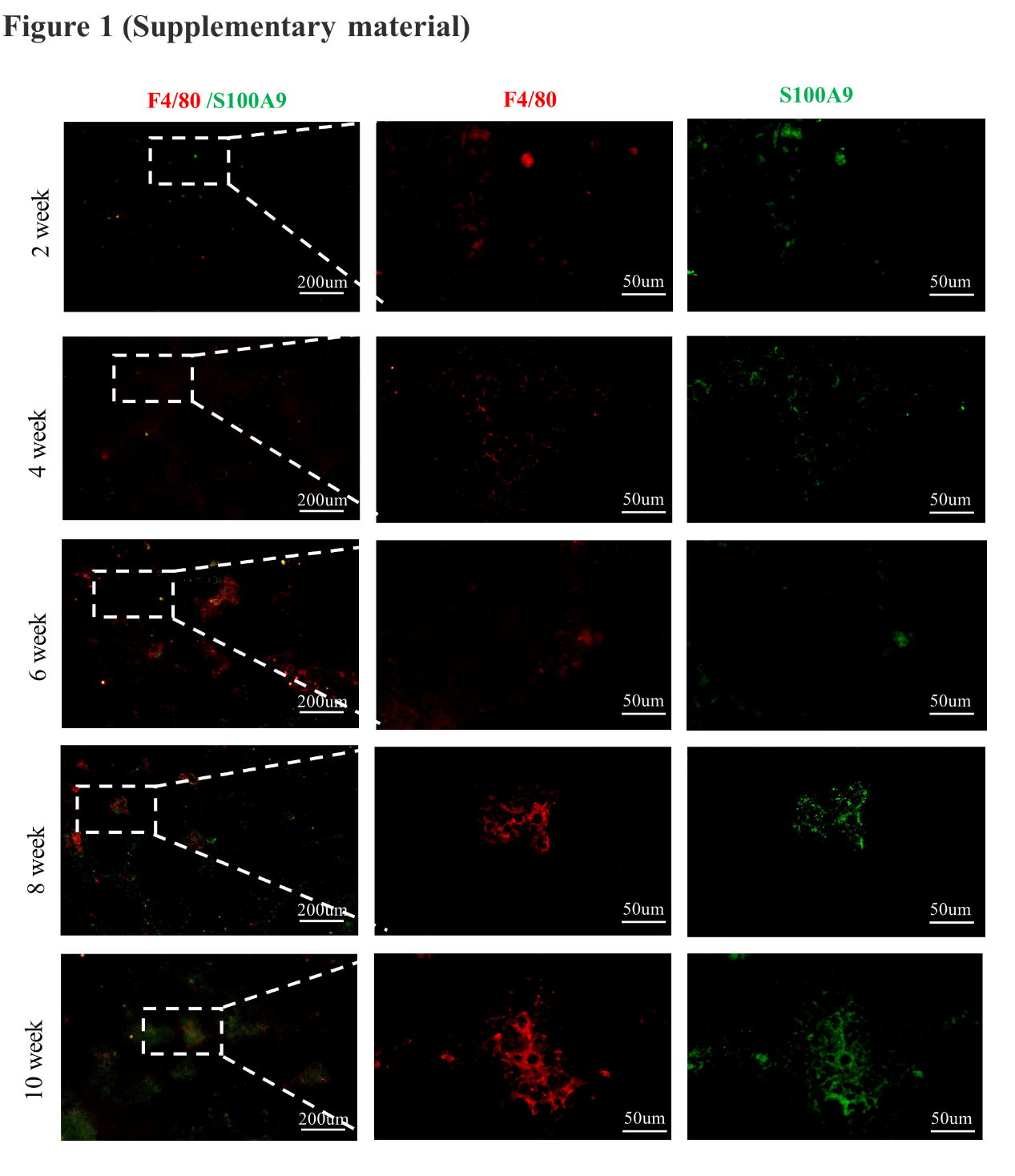
**

**Supplementary Figure S1**

Double immunofluorescence staining of S100A9 and macrophage marker (F4/80) in testis from weeks 2 to 10 (scale bar: 200 and 50μm) in **Figure 1**.

**
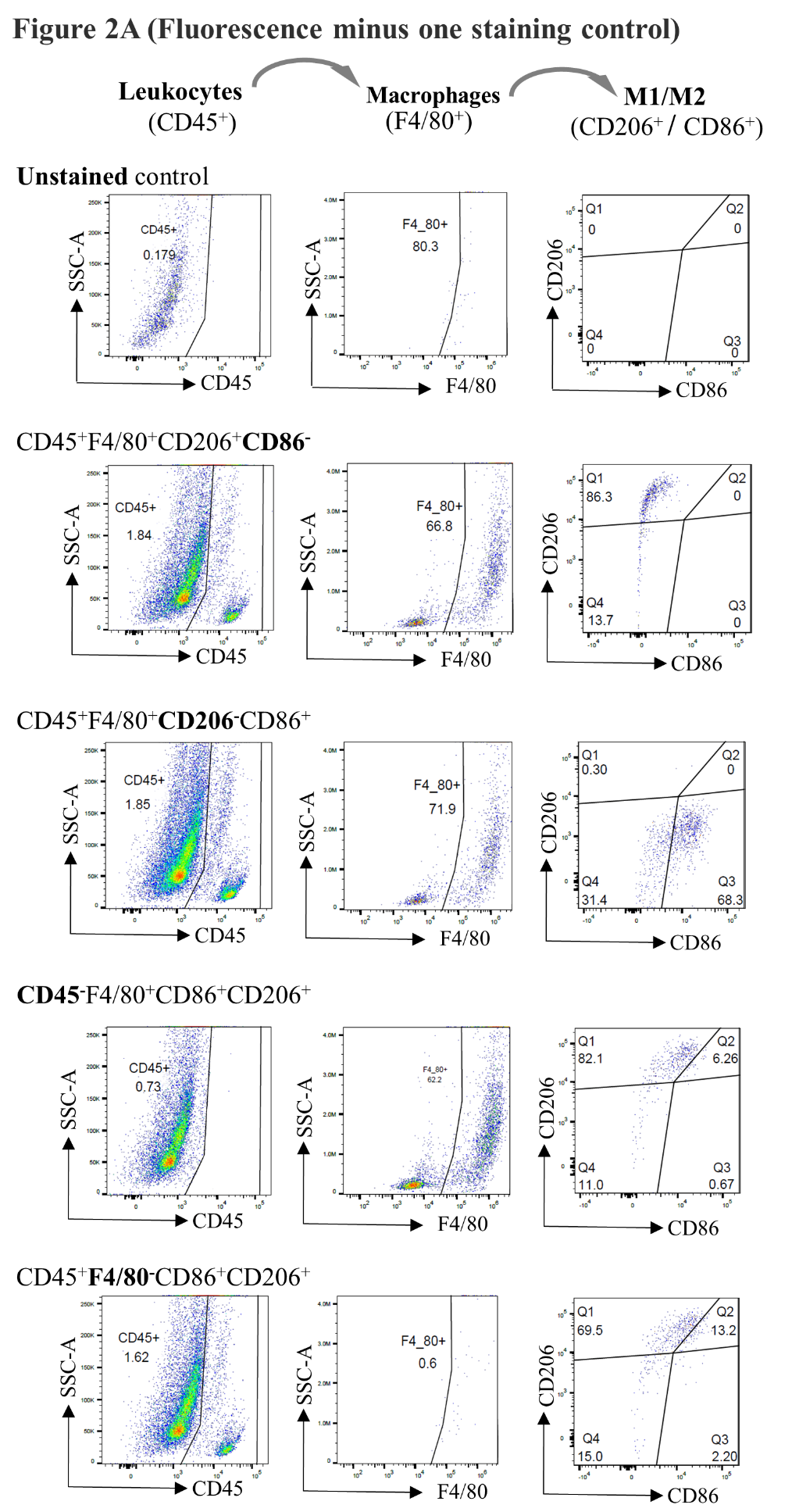
**

**Supplementary Figure S2**

The FMO (fluorescence minus one) control of each fluorescent marker in **Figure 2A**.

(8week, n=2).

**
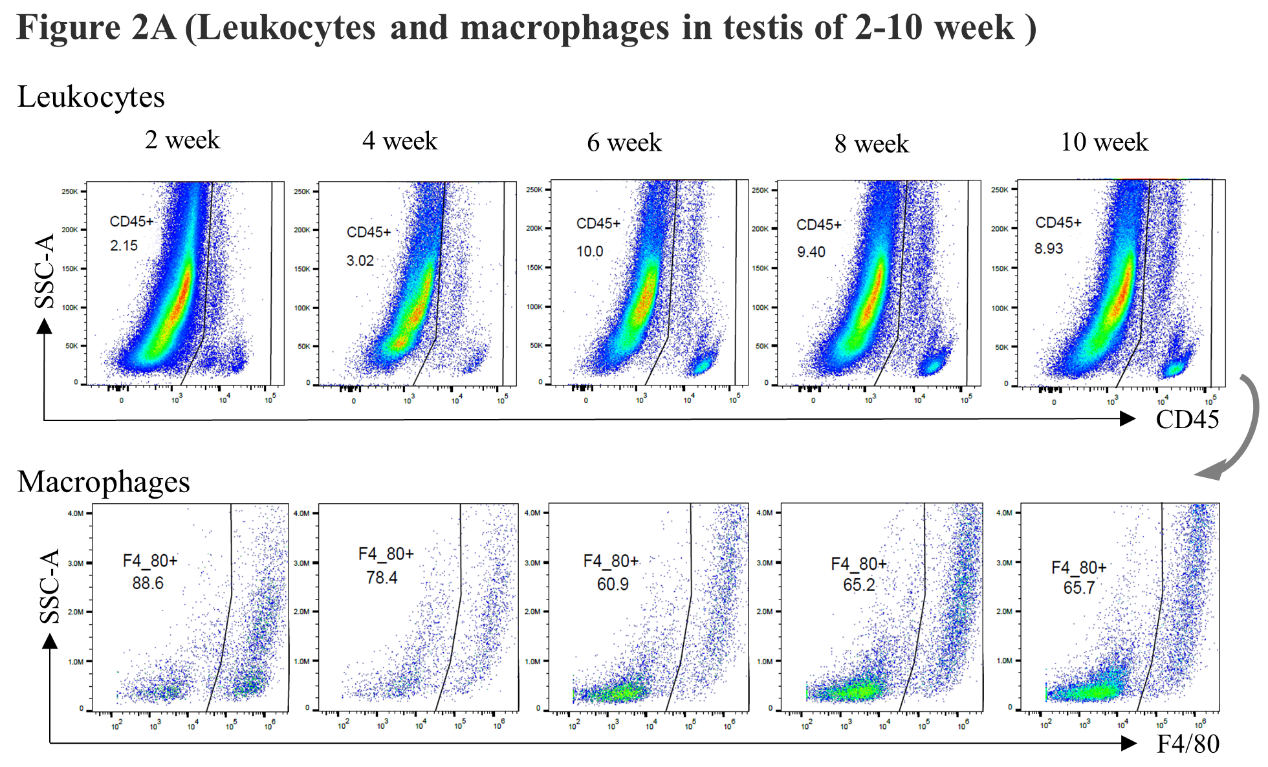
Supplementary Figure S3**

Percentage of leukocyte (CD45^+^), macrophage (CD45^+^F4/80^+^) in different age of testis by FCM (2week, *n*=4/group; 4-6week, *n*=3/group; 8-10week, *n*=2/group).

**
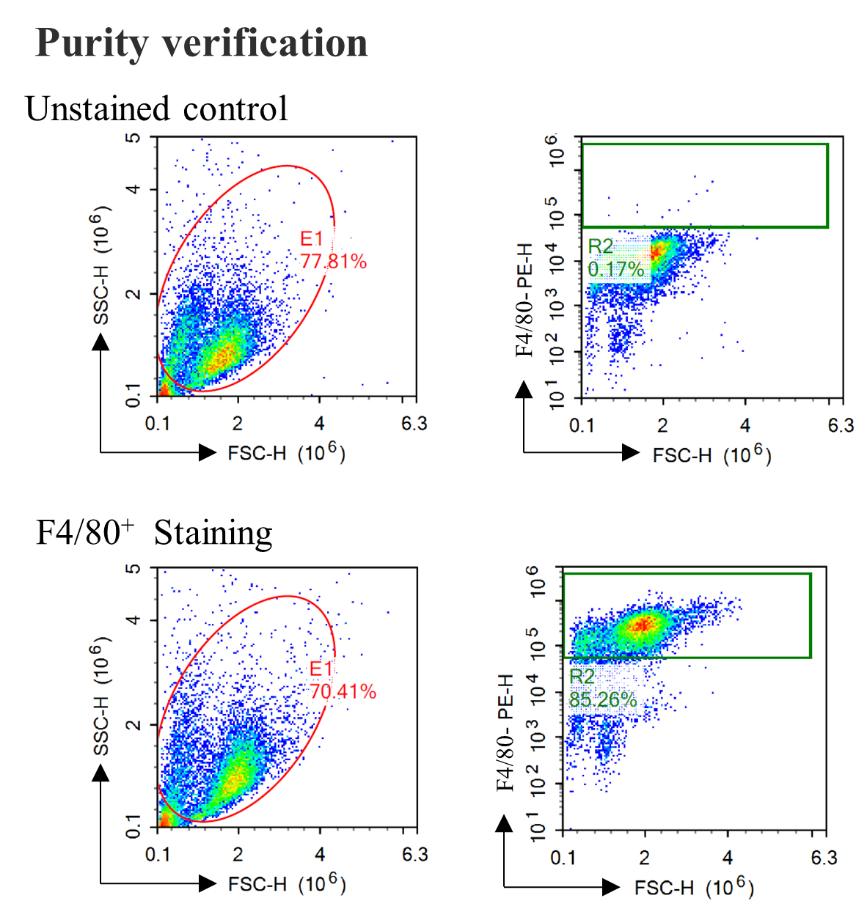
**

**Supplementary Figure S4**

The purity of TMs (8week, *n*=4/group) was verified using F4/80 antibody.


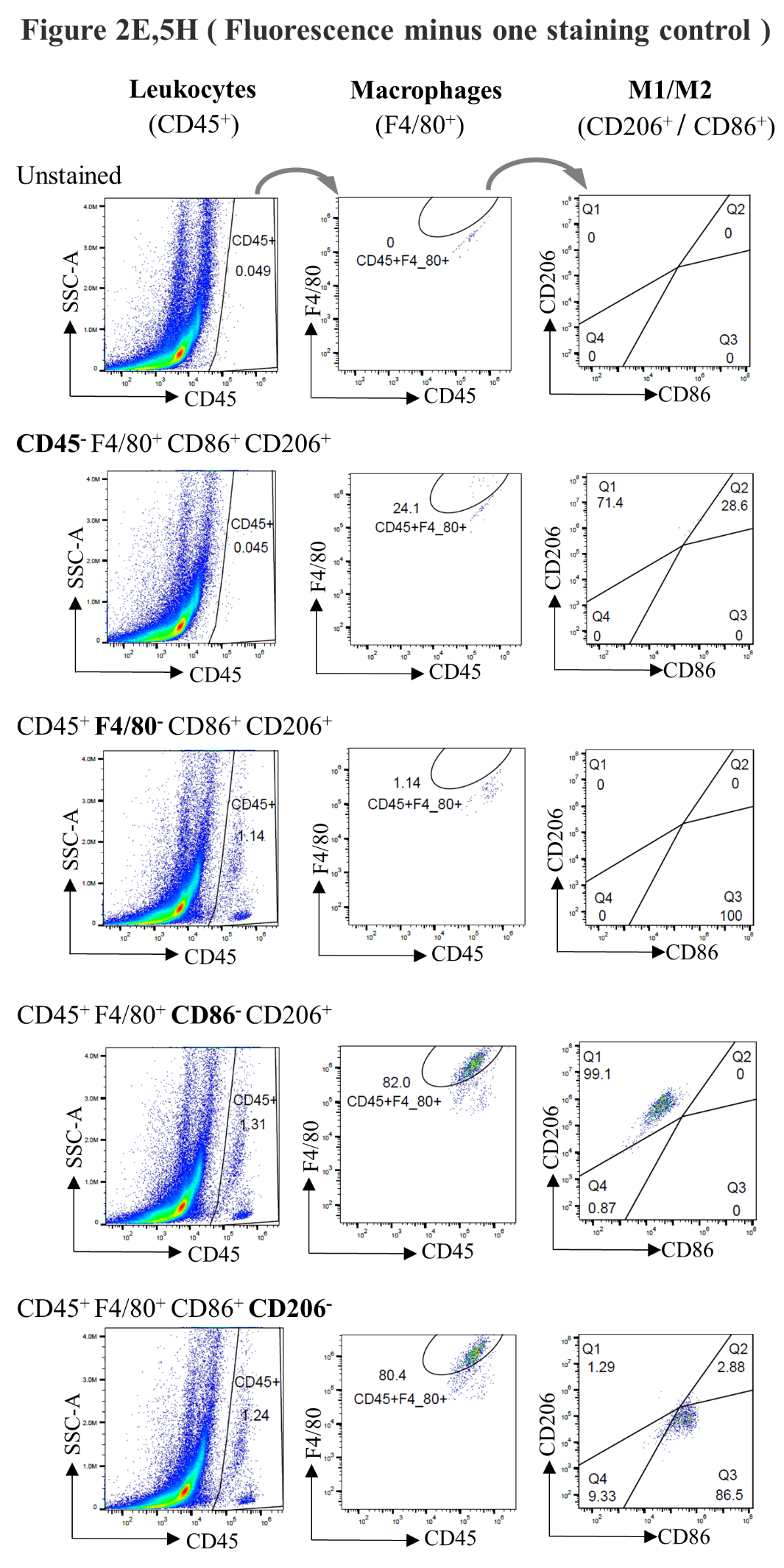


**Supplementary Figure S5**

The FMO (fluorescence minus one) control of each fluorescent marker in **Figure 2E and 5H**. (8week, n=4).

**
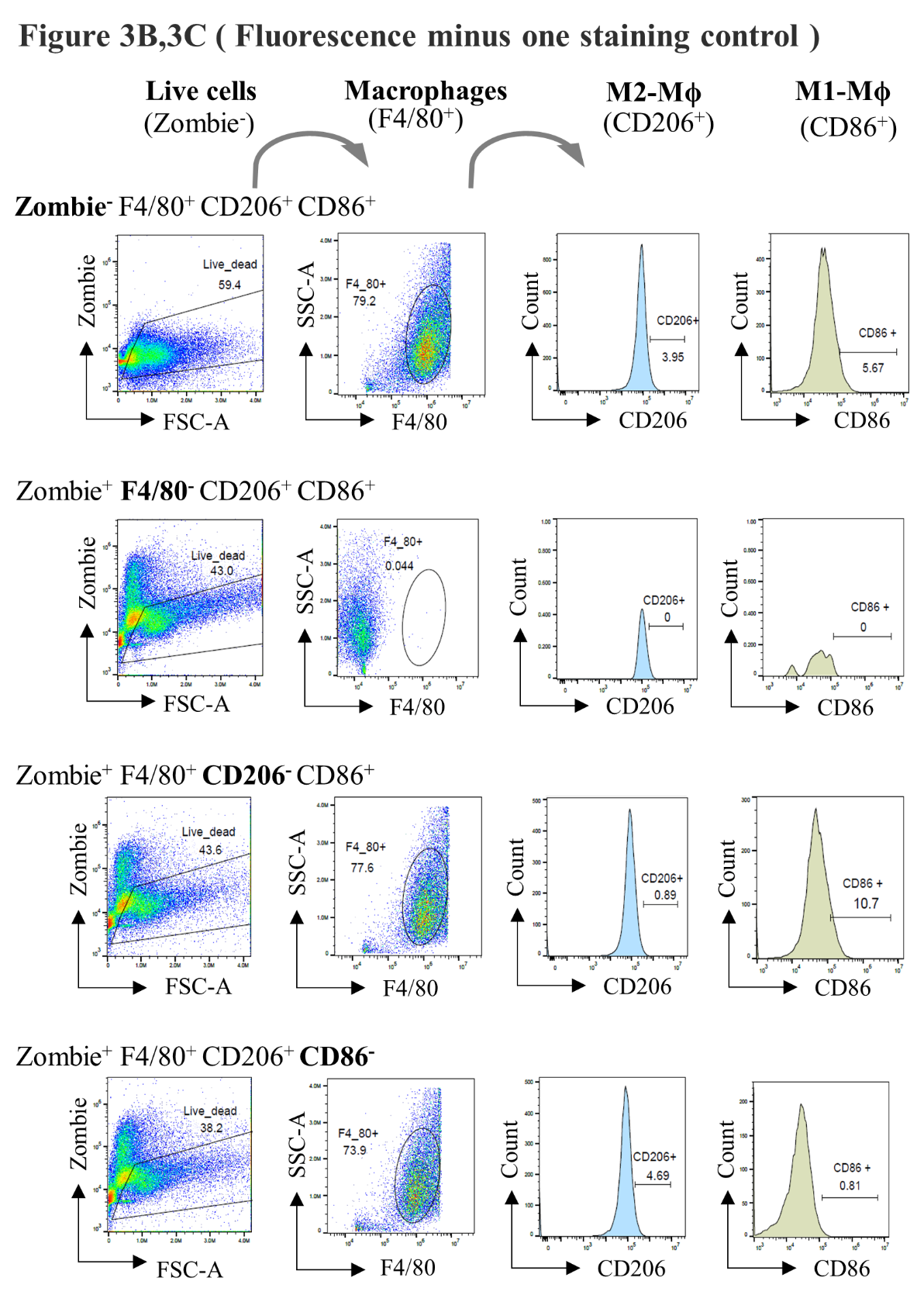
**

**Supplementary Figure S6**

The FMO (fluorescence minus one) control of each fluorescent marker in **Figure 3 (B and C)**. (6week, n=4/group).

**
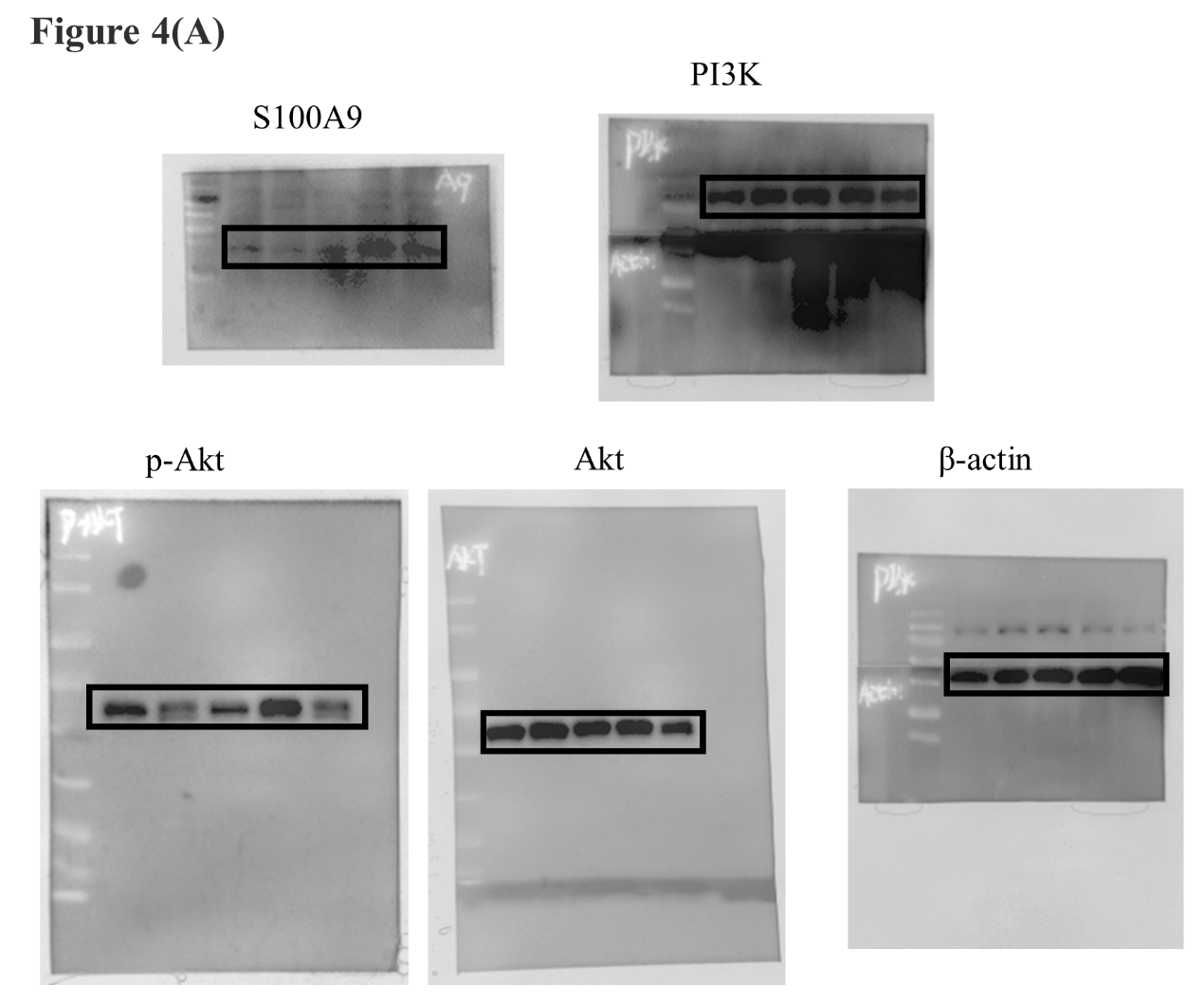
**

**Supplementary Figure S7**

Bands were detected using anti- S100A9, anti-PI3K, anti-p-Akt, anti-Akt and anti-actin antibody in Raw 264.7 macrophages **in Figure 4(A)**.

**
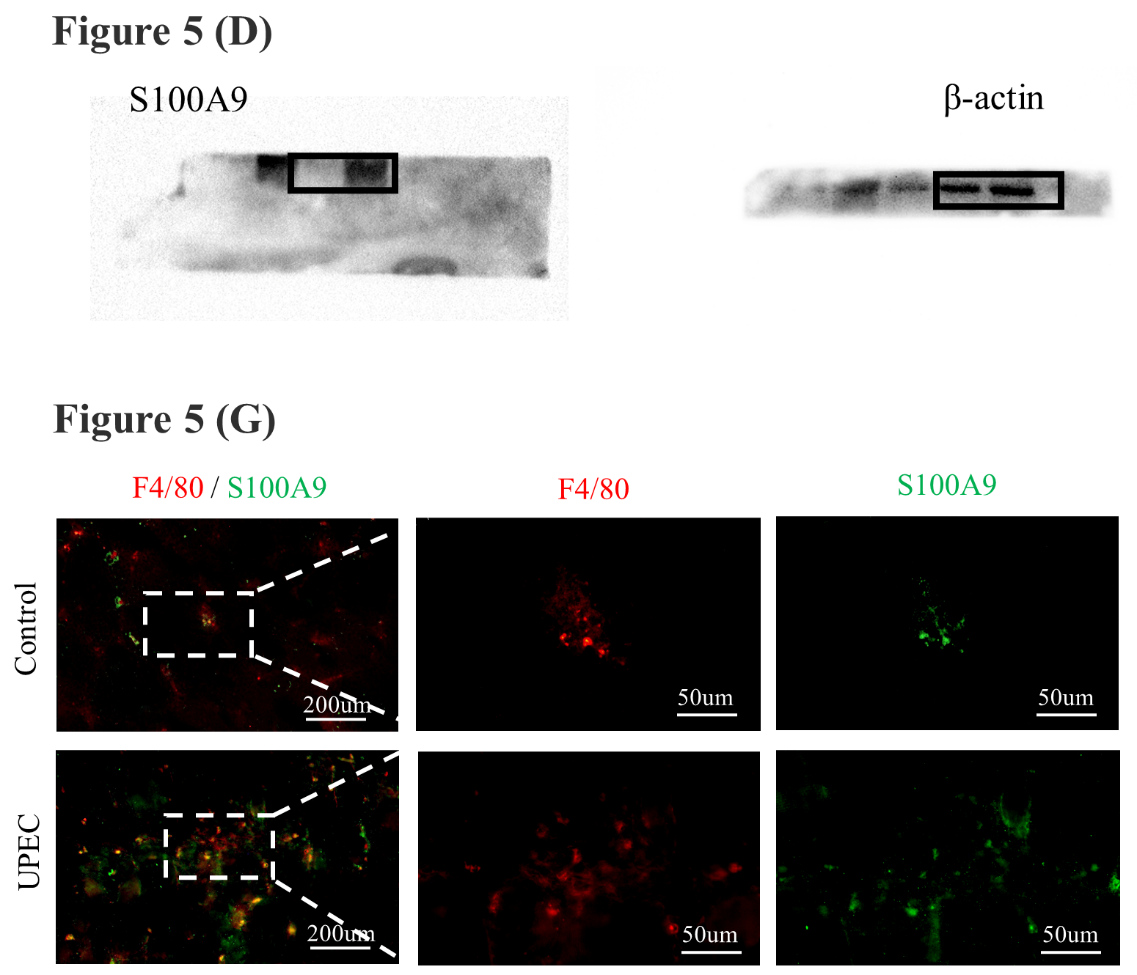
**

**Supplementary Figure S8**

Bands were detected using anti- S100A9 and anti-actin antibody in testicular macrophages **in Figure 5D**. (8week, n=4/group); Double immunofluorescence staining of S100A9 and macrophage marker (F4/80) in testis of control and UPEC (scale bar: 200 and 50μm) in **Figure 5 (G)** (8week, n=2/group).

**Supplementary Table S1**

**Primers and sequences used in Plasmid construction.**

1. **Construction of overexpression vectors**

| Primer name | Primer sequence 5’to 3’ |
| --- | --- |
| Primer-F | CGCGAATTCGAAGTATACCTCGAGGCCACCATGGCCA |
| Primer-R | CGATCGCAGATCCTTGGATCCTTACTTCCCACAGCCTTTGC |

**
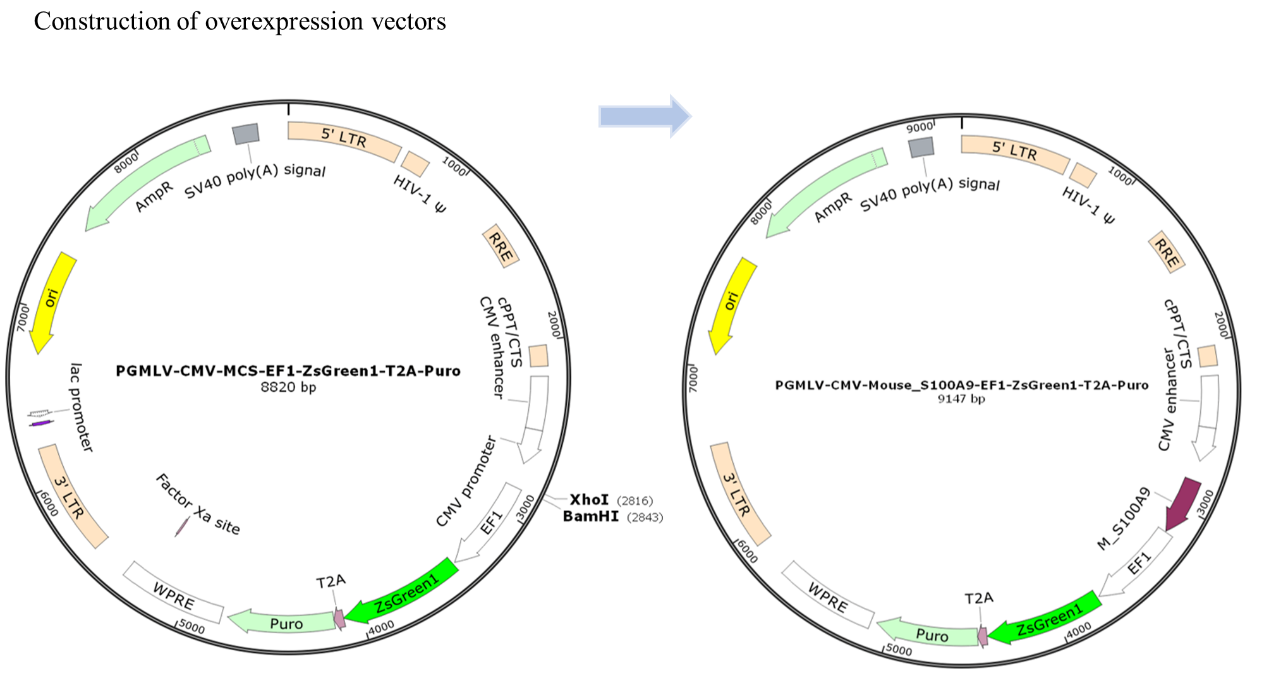
**

1. **Construction of three shRNA vectors**

| **NO.** | **TargetSeq** |
| --- | --- |
| Negative Control (NC) | TTCTCCGAACGTGTCACGT |
| 1 | GCACAGTTGGCAACCTTTATG |
| 2 | GCCCTCATAAATGACATCATG |
| 3 | GGACAATCAGCTGAGCTTTGA |

**
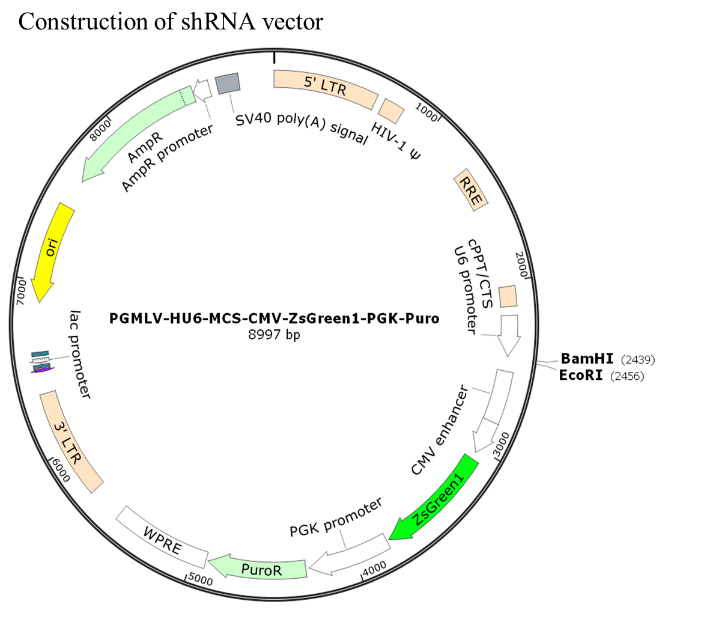
**

**shRNA DNA sequence**

| Oligo name | DNA sequence 5’to 3’ |
| --- | --- |
| Primer-NC-T | gatctGTTCTCCGAACGTGTCACGTTTCAAGAGAACGTGACACGTTCGGAGAATTTTTTc |
| Primer-NC-B | aattgAAAAAATTCTCCGAACGTGTCACGTTCTCTTGAAACGTGACACGTTCGGAGAACa |
| Primer-T1 | GATCCGCACAGTTGGCAACCTTTATGCTCGAGCATAAAGGTTGCCAACTGTGCTTTTTT |
| Primer-B1 | AATTAAAAAAGCACAGTTGGCAACCTTTATGCTCGAGCATAAAGGTTGCCAACTGTGCG |
| Primer-T2 | GATCCGCCCTCATAAATGACATCATGCTCGAGCATGATGTCATTTATGAGGGCTTTTTT |
| Primer-B2 | AATTAAAAAAGCCCTCATAAATGACATCATGCTCGAGCATGATGTCATTTATGAGGGCG |
| Primer-T3 | GATCCGGACAATCAGCTGAGCTTTGACTCGAGTCAAAGCTCAGCTGATTGTCCTTTTTT |
| Primer-B3 | AATTAAAAAAGGACAATCAGCTGAGCTTTGACTCGAGTCAAAGCTCAGCTGATTGTCCG |
